# Understanding acceptance of and adherence to a new formulation of paediatric antiretroviral treatment in the form of pellets (LPV/r)—A realist evaluation

**DOI:** 10.1371/journal.pone.0220408

**Published:** 2019-08-21

**Authors:** Ariadna Nebot Giralt, Christiana Nöstlinger, Janice Lee, Olawale Salami, Marc Lallemant, Washington Onyango-Ouma, Isaac Nyamongo, Bruno Marchal

**Affiliations:** 1 Department of Public Health, Institute of Tropical Medicine, Antwerp, Belgium; 2 Drugs for Neglected Diseases Initiative, Geneva, Switzerland; 3 Drugs for Neglected Diseases Initiative, Nairobi, Kenya; 4 Institute of Anthropology, Gender and African Studies, University of Nairobi, Museum Hill, Nairobi, Kenya; University of South Florida, UNITED STATES

## Abstract

**Background:**

Improving access to paediatric HIV treatment requires large-scale antiretroviral treatment programmes and medication adapted to infants and children’s needs. The World Health Organisation recommends lopinavir/ritonavir plus two nucleoside reverse transcriptase inhibitors as first-line treatment for all HIV-infected children younger than three years, usually given as a syrup. A pellet formulation (i.e. tiny cylinders of compressed medication put in capsules) was developed to overcome the syrup formulation’s disadvantages such as bitterness, toxicity and cold storage. This study assessed multi-level factors influencing caregivers’ acceptance of and adherence to lopinavir/ritonavir pellets as well as their underlying mechanisms.

**Methods:**

A realist evaluation (a theory-driven evaluation method considering the social context and mechanisms of change), embedded in a clinical trial was carried out in three hospital settings in Kenya. Data were collected through document review, observations (n = 34) in home and clinic settings and semi-structured interviews (n = 44) with caregivers and providers. Data analysis was based on realist principles.

**Results:**

High levels of treatment initiation and adherence were observed. Taste masking, neutral packaging and easy storage made the new formulation highly acceptable. Caregivers developed individual strategies to deliver the treatment, particularly to overcome specific problems e.g. in case of just-weaned babies or food shortage. A refined program theory emerged from the triangulated findings showing that ease of administration combined with increased self-efficacy and competences of the caregivers, and effective provider support contributed to high levels of adherence.

**Conclusions:**

Formulating combined antiretroviral treatment in the form of pellets is clearly a more acceptable solution for infants and children and their caregivers compared to the syrup. Further research in non-trial settings may shed light on factors related to providers, services and the health system that contribute to better adherence of such formulations.

## Introduction

Much progress has been made in the field of paediatric antiretroviral therapy (ART), but major treatment gaps persist. In 2017, only 52% of children under 15 years of age living with HIV were on antiretroviral treatment [1]. Only half of the HIV positive infants were diagnosed and this hampers immediate initiation of ART as recommended by the World Health Organization (WHO) [2,3,4].

The large-scale introduction of Highly Active Antiretroviral Therapy (HAART) in the 1990s significantly reduced AIDS-related mortality [5]. Optimal adherence is the most important determinant of effective ART, and at the same time one of the major challenges for paediatric HIV care and treatment programmes [6]. WHO suggested that rates of adherence should exceed 95% to continuously control HIV replication and maximize the potential benefits of ART [2]. The use of lopinavir/ritonavir (LPV/r)plus two NRTIs has been recommended by WHO in 2013 for first-line treatment for all children infected with HIV younger than three years of age [7]. However, administering LPV/r to infants and young children is difficult: While LPV/r syrup can be given to children with difficulties to swallow tablets [8], it has a very bitter taste and contains high concentrations of alcohol and solvent. It must be stored in a refrigerator or below 25°C.

New formulations of LPV/r have been developed in response. Cipla Ltd., an Indian pharmaceutical company, has improved this formulation, assembling LPV/r in the form of mini tablets or pellets, of which 43 are put in a capsule that can be opened to administer the pellets with breast milk, formula, other liquids or small amounts of solid food. Because this formulation tastes better and is easy to store and transport, it has been found more acceptable [9]. The LPV/r pellet formulation received tentative approval by the United States Food and Drug Administration (FDA) in 2015.

Drugs for Neglected Diseases initiative (DNDi) conducted a study to assess safety and effectiveness of this formulation in 12 clinical trial sites in Kenya, Uganda and Tanzania [10]. The objective of the LIVING study (registration number NCT02346487) was to evaluate the effectiveness of LPV/r pellets in addition to nucleoside reverse transcriptase inhibitors (NRTIs), i.e. ABC/3TC (abacavir/lamivudine) or AZT/3TC (zidovudine/lamivudine) dispersible tablets under routine treatment conditions in HIV-infected infants and young children who cannot swallow tablets. Acceptability of the pellets and their ease of use for caregivers and health care workers were included as secondary endpoints. As it is the first time that such formulation will be made available in ART programmes in sub-Saharan Africa and in order to inform the scaling up, DNDi commissioned a realist evaluation to provide more insights into the underlying mechanisms leading to acceptance and adherence. This study—the RE-LIVING study [11]—was conducted in 3 sites in Kenya, with the aim to specifically explore caregivers’ acceptance of and adherence to the new formulation, and how this may be related to the new formulation’s acceptability, i.e. the pellets’ product characteristics. We present the results in this paper.

## Methods

**Fig 1:**
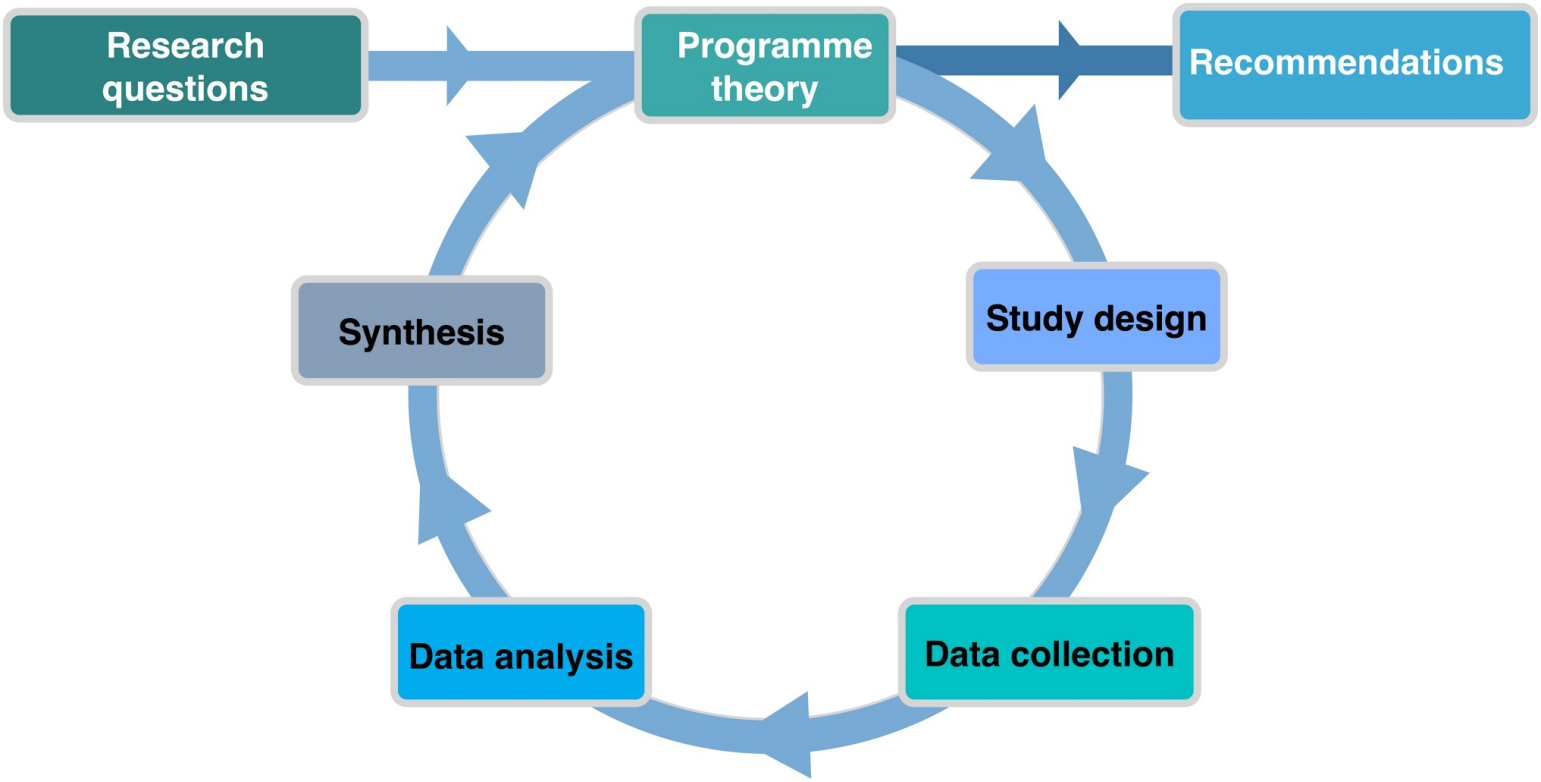
The realist cycle [13].

Given the exploratory nature of the study, we adopted the realist evaluation (RE) approach [12]. RE belongs to the school of theory-driven inquiry, and argues that evaluation research needs to answer the questions ‘what works in which conditions for whom’, rather than merely ‘does it work?’ to produce useful results for decision makers. RE considers that interventions work (or do not) because actors may or may not take up what is provided by the intervention. The interaction between ‘intervention’ (here referring to LPV/r pellets delivery and health care provider support to administer them correctly and consistently within the LIVING trial) and ‘actors’ in specific ‘contexts’ triggers ‘mechanisms’ that cause ‘outcomes’ to occur. A realist evaluation thus seeks to identify the configuration of factors related to the formulation, the illness, the caregivers, and the setting that explain how this formulation may be more acceptable (or not). In other words, it opens the black box between the intervention (in casu the pellet formulation) on one hand, and the outcomes (acceptability and adherence) on the other. In RE, the analysis explicitly builds upon existing knowledge and evidence. Indeed, a realist evaluation starts from a hypothesis which is called the initial programme theory. A programme theory can be defined as the (hypothetical) understanding of how a programme or intervention is expected to lead to its intended effects, for whom, in which conditions and how. It can be constructed on the basis of literature reviews, exploratory research and project document reviews. The IPT is in fact a hypothesis that is tested through empirical research. The end result of a RE is a refined programme theory which may inform further trials elsewhere with similar or other pellet formulations. A detailed presentation of the RE approach can be found in the published study protocol [11]. Fig 1 presents the realist research cycle, along which we structured our study.

### Eliciting the initial programme theory

As required by the RE approach, we started with eliciting the initial programme theory. To do so, we first carried out a review of the documents and protocols of the LIVING study. Through discussions with DNDi staff working on the LIVING study at the headquarters in Geneva and in Kenya, we obtained more insights into their assumptions regarding the causal processes underlying the new formulation’s potential effects. Finally, we carried out a narrative literature review on paediatric ART adherence, focusing first on reports and papers of clinical trials of the new formulation. In a second step, we reviewed behavioural science theories on adherence and acceptability [11].

**Fig 2:**
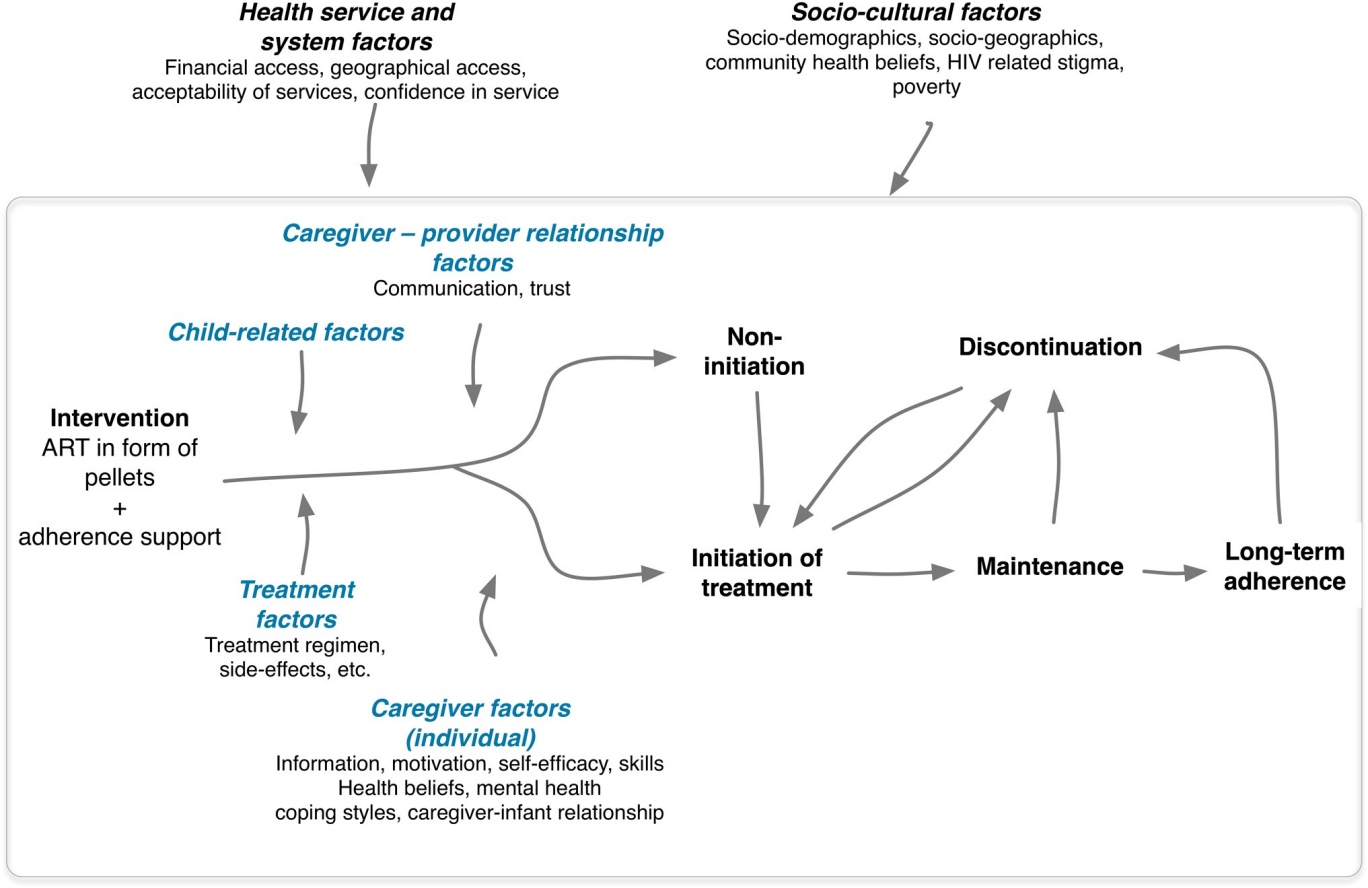
The initial programme theory.

Combining the insights gained from these approaches, we drafted the initial programme theory (Fig 2). In a nutshell, it stipulates that the individual pathway from intervention uptake to long-term adherence may initially be influenced by the pellet formulation’s acceptability, which in turn is affected by child-related (e.g. age, gender, ability to swallow) [14–16] and treatment-related factors (taste, storage) [16–17]. Since children are dependent of their caregivers to initiate treatment, caregiver-related factors come into play.

One of the empirically best validated individual-level theories designed to explain HIV prevention behaviour, i.e. the Information-Motivation-Behavioural skills model (IMB) [18], has been widely adopted to explain adherence behaviour. Therefore, our initial programme theory integrated the IMB model, stipulating that correct knowledge about the pellets, motivation to use them, and the necessary skills to administer them correctly may explain initiation. Self-efficacy is a central mechanism in the IMB model, i.e. the belief to be able to perform the specific behaviour, which is related to skills building and influences outcome behaviour. Health service- and systems factors (such as access and communication with service providers) may have an impact on all of the above-mentioned determinants through increasing knowledge and motivation and contributing to building the necessary skills. Likewise, similar factors at the caregiver-level may further determine if initial uptake will be maintained over time. However, from an ecological perspective, health-care provider factors (patient-provider communication) [6, 19], health service-related factors such as management style and organisational culture at the clinic [20–21], as well as community-related factors, including community health beliefs and HIV-related stigma [22] may additionally enhance or hinder the process from maintenance to long-term adherence. More details on how the initial programme theory was developed can be found in the published protocol [11].

### Study design and settings

We adopted the multiple embedded case study design [23]. We defined the case as the caregiver-infant couple or the extended family setting where paediatric HIV care takes place. We invited for participation caregiver-infant couples who were enrolled in the LIVING study taking the new pellet formulation. We purposively selected information-rich cases [24], based on criteria of age and socio-demographic background to achieve a maximum variation sampling. We sampled in the following three facilities to provide for potential contrasts and comparison, aiming at including 15 caregivers and five providers in each hospital.

- The Family AIDS Care and Education Services (FACES) project in Kisumu operates in the Lumumba Health Centre in Kisumu, serving an urban and peri-urban population.
- The HIV treatment programme of Kenyatta National Hospital (KNH) is one of the largest in the country. Most children attending the paediatric HIV clinic come from informal settlements in and around Nairobi.
- The Gertrude’s Children’s hospital in Nairobi runs a paediatric HIV treatment service free of charge. Over 60% of the children cared for come from informal settlements.

### Data collection methods, tools and procedures

We started with a document review and a narrative literature review. We adopted qualitative data collection techniques, i.e. semi-structured interviews (SSI) and participant observations (PO).

#### Document review

Documents related to the LIVING study were collected and analysed, including the intervention guidelines and implementation reports. This allowed to describe the intervention and its actual implementation in the three facilities, and contributed to formulating the initial programme theory. Final results of the LIVING study were not yet available at this stage of the research.

#### Semi-structured interviews

We carried out semi-structured interviews with caregivers of HIV positive infants and with health care providers in either English or Kiswahili, based on participants’ preferences. Interviews were conducted by trained social scientists using a pre-developed and pilot-tested topic guide in a flexible manner [25].

The topic guide for caregiver interviews consisted of eight topics, related questions and probes, in line with the initial programme theory. It aimed at capturing their personal background, children’s HIV status and experiences with paediatric ART, caregivers’ own HIV and health status, experiences with administering the pellets, beliefs related to the importance of adherence and motivations to adhere, detailed information on treatment initiation, continuation and potential discontinuation, specific barriers and facilitators to adherence including adherence support received, and caregivers’ future aspirations for their children and themselves.

The interview topic guide for health care providers focused on exploring their personal opinions, beliefs and perceptions of the acceptability of the pellets, the adherence support given, perceived provider-patient relationships and communication.

Candidate interviewees were personally invited to participate in this study by the LIVING study nurses. If interested, they received a participant information sheet explaining the study objectives and procedures, and the informed consent (IC) form was explained. The IC included approval to record the interview. Interviews were conducted in English or Kiswahili according to interviewees’ preference and the interviews were recorded upon agreement of the respondent. We interviewed a total of 41 caregivers and 12 providers (Table 1) between 01–09/2017, which was sufficient to achieve data saturation.

**Table 1:** Overview of interviews and observations per site.

		Kenyatta National Hospital (KNH)	Gertrude’s children hospital (GCH)	Kisumu Faces clinic (FACES)	Total
Semi-structured interviews	Providers	4	4	4	12
	Caregivers	13	15	13	41
Clinic Observations		4	9	4	17
Home Observations		3	10	4	17
**Total**		**24**	**38**	**25**	**87**

#### Participants characteristics

Table 2 presents some key characteristics of infants and children included in the study. The majority of the children were aged between two and four years, and in most cases the mother was the primary caregiver.

**Table 2:** Demographic characteristics.

		Total
Infants	Male	20
	Female	21
Age	Less than 1yr	0
	1 yr	8
	2 yr	14
	3 yr	8
	4 yr	7
	5 yr	3
	> 5 yr	1
Orphan	Single (father had died)	5
	Double	0
Primary caregiver	Mother	37
	Father	1
	Grandmother	3
Primary caregivers’ education level	Lower than Primary school	3
	Primary school	13
	Secondary school	10
	Higher education	4
	College/university	10
	Other	1

#### Participant observation

We conducted participant observations (PO) at the clinics and in study participants’ home environments. We used PO as an overt, complementary method to validate participants’ reporting of what they belief and do [26]. At the clinic setting, observations focused on the interaction and communication between providers and study participants. In the home environments, we mainly observed how study participants gave the pellets to their children, the caregiver-infant interaction, the interaction with family members and significant stressors present in the home environment and available support mechanisms. Home observations provided an impression of participants’ current living situation. POs were conducted systematically using pre-defined checklists and were followed by informal unstructured interviews. The voluntary nature of accepting the home visits was emphasised to all study participants.

### Data analysis

All recorded interviews were transcribed verbatim and translated. Transcripts were checked for accuracy at random and adapted, if necessary. All transcripts, related memos and PO field notes were entered in NVIVO 11 for analysis. For practical reasons (time, frequency of clinic visits, distance of homes from the hospital), it was not possible to provide the transcripts nor the results to the respondents for feedback.

The analysis consisted of two stages. After familiarisation with the data, we first coded the data based on the key elements of the initial programme theory. This resulted in an initial data-driven code-book. During a data analysis workshop with all involved researchers, we refined the code-book based on joint readings of several transcripts. Subsequently, all transcripts were coded, and categories and themes were refined iteratively in the process.

In the second stage of the data analysis process, we applied the retroduction approach, whereby the analysis aimed at explaining the observed outcomes according to the RE approach. In practice, the steps we followed were describing the significant outcomes and the actual intervention, retroduction to possible causal mechanisms, elimination of alternatives and identification of the generative mechanism(s). The Intervention-Context-Actor-Mechanism-Outcome configuration (ICAMO) was used as a heuristic to explore for patterns underlying acceptance and adherence [27]. There is still a lot of debate about what a mechanism is. We considered mechanisms as the causal pathways that explain how the intervention leads to an observed outcome in a particular context. More specifically, we followed Pawson and Tilley’s definition: an intervention triggers a mechanism that can be understood as actors’ choices in the sense of reasoning in response to the resources provided by the intervention. The ICAMO configuration builds upon the classic CMO configuration coined by Pawson and Tilley. By adding the ‘A’ for actors and the ‘I’ for intervention, the analyst is stimulated to analyse the data in order to explain how the observed outcomes are caused by a mechanism which is triggered by the intervention for specific actors in a specific context. For example, we matched the data categorised under ‘new formulation’ (intervention), ‘acceptance’ (outcome) with ‘caregiver factors’ (actor), ‘patient support’ (mechanism), and the Kenyatta Hospital setting (proximal context). During this process, new interpretations emerged in subsequent rounds of coding, leading to a refined analysis. The NVIVO database was made accessible to all researchers to contribute to this step. All data were anonymised and no personal identifying information was kept in these records.

We adopted several practices during the research process to maintain rigour and ensure trustworthiness of the analysis. Indeed, all along the process, the RAMESES II reporting standards for realist evaluations [28] were followed and as well as the COREQ checklist [S1 Checklist]. First, the use of theory, and specifically the initial programme theory, anchored the study in the existing body of knowledge. Purposive case selection ensured that the IPT could actually be tested. We attempted to sample as many caregivers as possible and to have respondents from different categories of patients. We organised a data analysis workshop in Nairobi with the research team to compare our approach to coding and to set up the initial coding tree. Triangulation was applied in a number of ways. Data from different sources (interviews, observations, document reviews) were triangulated during the analysis. We compared the patterns we found in terms of ICAMO configurations across all sites. Finally, the workshop and skype meetings with the research team allowed us to triangulate between the different research perspectives. Indeed, the multi-disciplinary nature of the research team, comprising 2 anthropologists, 2 pharmacists, 1 psychologist, 1 public health specialist/medical doctor, and 2 epidemiologists allowed for rich exchange during the analysis.

### Ethical considerations

The RE-LIVING study was approved by The Institutional Review Board of ITM (Ref. IRB/AB/Ac/061), the Ethical Committee of the University Hospital Antwerp (UZA) (Ref. B300201628563), the Kenyatta National Hospital University of Nairobi Ethics & Research Committee (Ref. KNH-ERC/A/293), the Ethical Review Board of Gertrude’s Children Hospital (Ref. GCH/ERB/VOLMMXVII/113), and the KEMRI/Scientific and Ethics Review unit (Ref. KEMRI/RES/7/3/1).

The study was carried out according to the principles stated in the Declaration of Helsinki, all related applicable regulations and according to established international scientific standards. All members of the research team were aware of the potential sensitivity of adherence, particularly among members of poor and vulnerable communities. Therefore, voluntary study participation and confidentiality in data collection and data handling were strongly emphasised when informing study participants. Study participants received no remuneration for their participation, but received 500 Kenyan Shilling per LIVING study visit to cover for transport costs.

## Results

### The intervention

All procedures were highly standardised, because the introduction of the new treatment took place in the context of a trial. The intervention consisted of provision of anti-retroviral treatment in the form of LPV/r pellets used in combination with NRTI in dispersible tablets, combined with clinical history taking, clinical examination and taking a blood sample for laboratory tests. Extensive information on the LPV/r pellets was provided using visuals. Our observations and interviews showed that the trial staff followed the trial guidelines closely at all the three sites.

#### Administration of the new formulation

The trial staff recommended caregivers to administer the pellets mixed with porridge, yoghurt, milk or water. Providers informed caregivers that the pellets required a number of steps for correct administration:

The pellets are a little bit more complex [to administer], because you have to get the capsules and you have to open them; you have to prepare the food, you have to make sure the child takes in all the pellets. You have to give it yourself, especially for the younger children. You have to open their mouth and check whether they have swallowed all the pellets. So, the pellets need more supervision from the caregiver. It needs more attention compared to the syrup.

(IDI Nurse Counsellor GCHM)

Opening the capsules to release the pellets for mixing with food or fluid may create some handling problems. In practice, most respondents found it easy, and some respondents mentioned that their (older) children did it themselves.

Another thing with the pellets is that it is easy to work with, because the child knows how to use it. She just takes the capsule, opens it and she takes the pellets with the yoghurt.

(IDI Caregiver KNH)

Most caregivers found a way to effectively administer the pellets shortly after initiation of the new treatment. They figured out what worked best for them by trial and error, especially for younger infants who experienced difficulties to swallow big amounts of food or fluid.

So, I put some porridge in the spoon. I have a small bottle cap where I put the opened capsules. After I have poured all the pellets in the bottle cap, I place porridge in a spoon, then add the pellets on the porridge, and then I give it to him. You know he cannot swallow everything at once, so the few ones that remain in the mouth, that is when I ask him “Have you swallowed everything?”. If he says no, I give him more porridge to make sure he swallows.

(IDI Caregiver GCHM)

This ease of administration was confirmed by the POs at home: children generally did not protest and swallowed the drugs without problem.

Despite standardised information provided, some caregivers initially administered the drugs incorrectly, but eventually discovered the correct way.

(From Clinic observation notes)

I found out (the pellets) were very bitter if you crush it. So, it is better (…) to open the capsule, put some yoghurt first, then the pellets, then cover with some more yoghurt, then let her swallow it without crushing.

(IDI Caregiver KNH)

Respondents indicated that tailored support by health care providers played an important role in finding effective ways to administer the pellets.

I first gave the capsule with soda (soft drink), but he would spill the pellets. So, I started giving him with porridge. He first took it until he discovered something was in it, so he started refusing to take porridge. I then called the doctors to inform them that the child had refused to take mediation and they told me to continue trying: ‘He will eventually get used to it’. I tried, but he would vomit after taking it. So, I decided to dissolve the pellets in water, which he takes with ease.

(IDI Caregiver FACES KSM)

Our interviews with caregivers and the home observations showed that about half of the respondents mixed the pellets with yoghurt or porridge. The other caregivers used tea or water to disperse the pellets, or mixed the pellets with co-trimoxazole syrup. Both the interview and home observation data showed that especially poor caregivers found it important that the pellets could be administered with tea or water, since solid food was often considered expensive and not always available.

### Acceptance of the new formulation

Acceptance was high among both caregivers and health providers. Most caregivers found that the new formulation was easier to administer and to store compared to liquid formulation. This was confirmed by our POs performed at home.

Ok, my child was on the *Kaletra* syrup, and it was not easy at all to give her that medicine. Even her viral load was always detectable.

(IDI Caregiver GCHM)

Most of the providers were convinced that the new formulation was far better than the LPV/r syrup. Providers reported from their contacts with patients that storing and administering the LPV/r pellets was much easier compared to the LPV/r syrup. They also believed that the easy administration of the drugs to the children increased caregivers’ motivation to continue.

The fact that the child is getting the treatment without vomiting and spilling, and then the health progresses, that is what motivates them.

(IDI Nurse counsellor KNH)

We found some divergent opinions related to the taste of the pellets. While providers emphasised the virtual absence of bitterness, quite some caregivers mentioned that it was important to immediately give the pellets mixed with food or beverage to the child to avoid the development of a bitter taste. Most of them had found this out after having experimented a little while with the drug, which helped them to establish their own routine:

At first, I did not know how I will give him the drugs, because here, I was told to use water and in fact, it was hard for me to administer. I tried using water to administer the drug for one week and I saw that the pellets ended up remaining behind, and he [the child] cried. I tasted one and I found that the drug is really bitter. So, for yoghurt, when you dissolve it in it, he just swallows it and he does not feel anything at all. Even when you put it in porridge, it is okay.

(IDI Caregiver GCHM)

### Mechanisms underlying initiation and acceptance

We identified several mechanisms underlying the decision to switch to the new treatment and maintain it. They can be categorised in (1) caregivers’ own beliefs and (2) mechanisms triggered by the trial setting. The former included competence and autonomy, future aspirations related to children’s health, self-experience and perceived HIV-related stigma.

#### Competence and autonomy

Most respondents previously have had negative experiences with the syrup and readily saw the advantages of the pellets when staff explained how they can be stored and administered.

I like it because it is easy, no need to store it in a fridge or so. Again, when you place the medicines in his mouth, he will swallow. It is not like the syrup which he used to spit out, so it is good.

(IDI 012 Caregiver GCHM)

According to my experience, having used it for a while now, the new medication is good. I do not experience any stress with handling it, also it does not have any side effects on the child.

(IDI 01 Caregiver KNH)

#### Future aspirations related to children’s health

Some respondents felt that starting the new formulation was necessary for their child to survive. They expressed a sense of obligation linked to future aspirations and hope for the child’s healthy development.

I want my child to have good health and I would love to see him grow up.

(IDI Caregiver KNH)

I said to myself that I did not have an alternative. I would therefore have to give her medication.

(IDI Caregiver FACES KSM)

#### Self-experience

Some respondents living with HIV who had already experienced the positive effects of antiretroviral treatment for themselves immediately understood the advantages of the new formula and quickly developed a routine for administering the drugs:

I witnessed my own parent die of HIV. My mum used to use the very first drugs that were given to people with HIV. But my mum died and my older sister also died of the virus and she was looking very bad. Just because they did not strictly adhere to the rules of taking the medication well. (…) So, I realised that if you adhere to this medication and ensure that you follow the rules and regulations for taking this medication, you will be ok.

(IDI Caregiver KNH)

#### Perceived HIV-related stigma

Perceived HIV-related stigma emerged from our data as an important contextual factor influencing decisions and behaviours of caregivers in relation to treatment initiation. It subsequently also influenced how caregivers administered the LPV/r pellets. During a home visit, for instance, it was observed how a caregiver hid in another room to administer the medication to her child, because she had a relative visiting.

Caregivers who had not disclosed their HIV status to anybody else than health care providers reported higher levels of HIV-related stigma than those who access to social support related to giving the medication. In particular, single women living in precarious conditions talked about enacted stigma in their personal environment (e.g. neighbours, relatives). They lacked social support networks to overcome the fear and the consequences of stigmatisation.

I have no one. Because, even my brothers whom I told of my status as being HIV positive, they only loved me for about a month with my child. After that they rejected me completely. Even at this very moment, if I was to call them and tell them that I have been locked out of the house for not paying my rent, they won’t help me (…) They rejected me completely and yet, I do not have parents whom I could leave my child with.

(IDI Caregiver KNH)

#### Organisational culture

Our initial programme theory would have predicted that management style and organisational culture of the clinic to play a role in influencing treatment initiation. However, we found that the strict adherence to the LIVING trial procedures overruled pre-existing practices and potential influences of the organisational culture.

### Specific mechanisms underlying adherence

#### Practical issues (ease of administration, storage)

Caregivers who perceived the process of administering the pellets as easy and observed no side effects reported that the whole process was less stressful for them.

They are not fighting while they are giving drugs, the child does not run away when she sees the mum is preparing medicine. At least, medicine administration is not an experience that you don’t look forward to in the morning and in the evening.

(IDI Clinical officer KNH)

Because it became easier, she is not disturbing me anymore. She simply takes the medication without vomiting. So, it gave me the strength to work hard and give her the medication as I was instructed by the doctor.

(IDI Caregiver GCHM)

The fact that cold storage was not required reduced stress and HIV-related stigma in multiple ways: for instance, caregivers no longer needed to worry about paying a storekeeper to keep the drugs cool nor about having to disclose the status of their child to neighbours.

… what used to stress everyone was that you needed a fridge to store the syrup, but not everyone can afford to purchase a fridge. So, this is the best drug as you don’t need a fridge.

(IDI Caregiver GCHM)

#### Accessing informal support

Being able to talk about HIV outside the medical settings was perceived as facilitating good adherence. Being supported by family members helped in administering the drugs at the right time, and contributed to reducing stress. It helped caregivers to cope in a positive way with adherence and HIV in general.

My sister … encouraged me, she called me to her place and I showed her the medication and in fact she was shocked, but she … told me I should not be afraid, they are with me and I should just be strong. They are there for me when I feel depressed and stressed.

(IDI Caregiver GCHM)

For some, informal peer support was a strong motivator for adherence. Discussing and sharing HIV-related problems with other caregivers in the same situation was perceived as providing good solutions.

I ask other parents about how their children respond when they are being given the medication, just to know if it is only my child who disturbs when being given the medication. We compare how our children behave … in a way, we advise each other.

(IDI caregiver, FACES)

#### Establishing a personal routine

Some respondents initially mentioned that they did not feel confident to be able to maintain adherence over time, but it took them a few weeks to develop their own routine. Important in this process was the specific support received by the providers, which helped caregivers to develop a sense of self-efficacy, slowly growing into establishing the required routine.

The confidence I had is that I was really close to the doctors and they gave me a lot of support. What encouraged me so much is how they came in and the research and tests they did.

(IDI Caregiver KNH)

Most caregivers developed active coping strategies to remind themselves to administer the treatment every day at the correct time. The most common individual practical tools included phone reminders, an alarm, a clock, or the time announced on the radio.

I set an alarm twenty minutes before the time to remind when it is almost time for my child to receive medication. I also set it early so that it gives me time to prepare the porridge or if I am going to use the yoghurt, I am able to go and buy it within this time.

(IDI Caregiver KNH)

Some of the study participants relied on integrating the administration of the pellets into the daily routine, thereby creating an association of actions, as for instance preparing food or leaving for work.

When I am cooking, I know it is a must that I give him his medication, then he will be able to eat this food that I am preparing for him.

(IDI Caregiver GCHM)

### The refined programme theory

**Fig 3:**
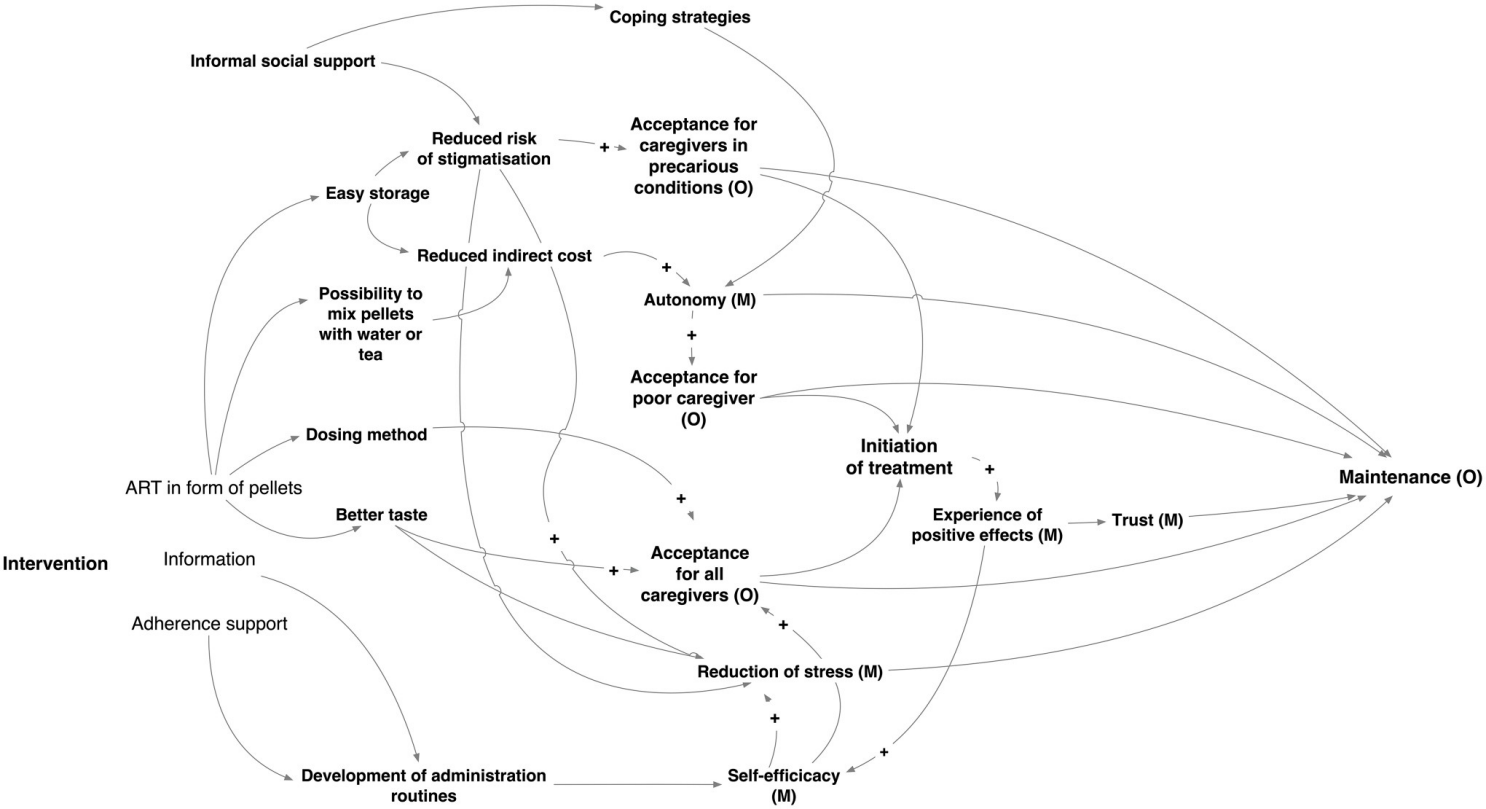
The refined programme theory.

These findings allowed us to refine the initial programme theory as depicted in Fig 3. The pathways of the initial programme theory were described in more detail and the influence of additional context-related determinants on initiation and adherence became apparent.

In presenting the key findings emerging from our data as ICAMO configurations, we mainly focus on factors that are modifiable and thus lead to relevant practical and clinical guidance in terms of how to support treatment initiation and adherence.

- In a context where the existing formulation has been very difficult to administer to young children (Context), caregivers (Actors) initiate the new treatment (Outcome) because of the good acceptability of the new formulation (Intervention), which is determined by its better taste, its packaging in smaller bottles, its ease of storage and its dosing method. These characteristics reduce stress during administration (Mechanism) and lead to caregivers’ acceptance of the pellets.
- In addition, several specific context-related mechanisms come into play: poor caregivers accept the new formulation because of the affordability of the administration: water or tea is sufficient to help the children swallow the pellets.
- Since its storage requires no refrigerator, the new formulation reduces the indirect cost and this enhances acceptability and user experience (i.e. acceptance). It thus increases poor caregivers’ autonomy.
- Since the formulation can be stored anywhere, it reduces the risk of unwanted HIV disclosure and stigmatisation, and thus lowers the secrecy and related stress.
- Caregivers who have experienced the benefits of ART, who are able to develop an active coping and problem-solving strategy to overcome initial problems with the new treatment, and who have future aspirations for their child(ren) and themselves, easily initiate and maintain the new treatment.
- Having experienced positive effects during the initiation phase of treatment (such as improved weight or health status of the child) increases caregivers’ trust into the product and increases their motivation.
- Developing practical and successful solutions for initial problems with administering the pellets increases caregivers’ self-efficacy, which in turn enhances the development of a daily routine and the maintenance of the treatment.
- Tailored and comprehensive professional adherence support increases caregivers’ self-efficacy, competence and autonomy by providing them with practical strategies that aim at developing a daily routine to administer the drugs, especially in the case of just-weaned babies.
- Informal social support in dealing with HIV-related problems at family-level or through peer support results in reduced levels of stress and in improved active coping with HIV related stigma, which in turn may promote adherence.

## Discussion

There is agreement in the literature that acceptability, while receiving an increasingly prominent role in implementation research, still is an ill-defined, poorly assessed and under-theorised concept [29]. In 2003, WHO published a framework for adherence that includes factors related to the specific therapy, the patient, the health condition, the health system, the health care team and finally the socio-economic context [30]. Useful as it is, this frame provides little explanation of the linkages and causal relationships between the factors in each and between each category and adherence–the framework is a-theoretical. The links between acceptability, treatment initiation, and short- and longer-term adherence need further exploration. In this study, we set out to explore caregivers’ acceptance of the new formulation and to understand how it affects adherence to treatment. The LIVING study’s interim results showed that 83% of the children were virologically suppressed at 48 weeks under the 2–1 treatment compared to 55% [31–32]. Against this background, we adopted the realist evaluation approach to develop a good understanding of acceptance and adherence, exploring how the pellets were administered, and why and in which conditions caregivers initiated and maintained the new treatment to their children.

Adherence is a strong predictor to viral suppression and paediatric adherence in low resource settings has been described as particularly challenging [6]. Paediatric HIV treatment has to be administered physically by caregivers to their children, since they are too young to decide for themselves or to take the medication. Consequently, the caregiver-infant dyad was of central interest to our research, and caregiver-related factors, such as their knowledge, motivation and skills to administer the regimen are of crucial importance. Likewise, community-, health-systems- and structural factors affecting adherence must be viewed in relation to the caregiver. For this reason, we identified the Information-Motivation-Behavioral skills model [18,33] as an appropriate starting point for building our initial programme theory. Individual level theories such as the IMB intrinsically focus on micro-level factors. However, no single factor or set of micro-level factors will adequately account for explaining adherence behaviour. Resources, cultural and social factors like HIV-related stigma, social control, community health beliefs, etc. may also affect adherence behaviour directly or indirectly, as evidenced by our findings.

Our results are overall aligned with the assumptions set out by the IMB model. Effective information and treatment support by providers generally increased self-efficacy and competences of the caregivers, in line with the IMB model applied to adherence [34]. This contributed to initiating and maintaining the new treatment. Interestingly, we found that caregivers’ own adherence to HIV medication was a strong motivator for consistently administering the pellets. Comparing caregivers’ own adherence with those of their children has not been done frequently, but Byakika-Tusiime et al. found optimal adherence levels in both groups [35].

On the level of treatment-related factors, the taste masking made administering the treatment to children much easier, reducing the concomitant stress. For poor caregivers, the possibility to administer the drugs with water or tea instead of with the more expensive yoghourt or porridge was important. Other studies have shown the associations between food insecurity and poor HIV treatment outcomes including adherence [36]. Women’s agency for feeding choices is related to both economic issues and local practices, which should be considered in comprehensive counselling [37]. Such contextual factors are important and revealing the mechanisms of how they may impede behaviour change even if motivated caregivers are given the right information can contribute to improved adherence. Our case studies show that treatment-related factors of good packaging and easy storage helped caregivers who chose not to disclose the status of their child. As such, the new formulation helped them to avoid stigmatisation and exclusion, reported by our respondents and still very much an issue in Kenya as evidenced by other research [38–39].

The importance of receiving effective provider or social support (beyond being informed) also emerged from our findings. Indeed, adherence to treatment was generally good once caregivers had developed a routine to administer the drug. This corroborates the crucial role of habit forming for effective long-term adherence [40]. Routine strategies were developed more easily when providers and family members were supportive, which also contributed to developing an active coping strategy. The significant role of family support has been highlighted by previous research on paediatric adherence [41]. On the contrary, barriers were confirmed that may impede adherence relating to situational factors, such as anticipated fear to unintentionally disclose HIV because the medication [42–43].

We acknowledge several limitations. First, we only relied on self-reported adherence, which may be subject to bias. A second limitation was the lower than planned number of home observations. Despite giving consent for home visits and observations, some participants opted out or withdrew consent (four in KNH, two in GCHM and two in Kisumu). We suspect that anticipating stigma in the personal environment may have accounted for the withdrawals. Third, the LIVING study was conducted in a highly standardised context of a clinical trial. Potentially existing differences between the settings could therefore not be observed, and provider-related factors were similar across all settings. Clinical and counselling services were delivered in near ideal circumstances in all three sites. Consequently, we could not investigate the effect of management style nor of organisational culture on staff behaviours. The additional measures that were part of the intervention (i.e. transport fare, counselling and support) may also have contributed to adherence. Such a conducive environment may not be easy to replicate during a scaling-up phase under real life conditions, unless specific attention is being paid to staff competences, motivation and working conditions.

The relatively large number of researchers from different institutions involved in this evaluation helped to deal with the potential issue of researcher bias, which could arise from the involvement of DNDi staff in the evaluation. We can report that at no point any pressure was exerted from within DNDi on the researchers from IAGAS or ITM.

The study provides a starting point for testing our findings in other settings: from a realist perspective, subsequent studies could assess whether this or similar formulations would work in the same way in different settings. When scaling up such new formulations, it will be important to carry out similar studies in different clinical settings, with specific attention to whether less resourced settings allow for the same degree of provider support.

Based on our results, we draw some general recommendations, acknowledging the specific trial study contexts in which the intervention was carried out. While the pellets present major advantages over the previous syrup-based formulation, their better acceptability is in itself probably not sufficient to enhance optimal adherence. Good information about the pellet formulation and how it can be administered needs to be supplemented by caregiver-tailored support that aims at overcome their specific problems, especially in the case of caregivers with infants.

Introducing the pellets should focus on the benefits as perceived by the caregivers, on supporting them with problem-solving skills to establish their own routine, and identifying the contextual risks grounded in social factors and community norms potentially leading to treatment interruptions or non-adherence as identified in this study (e.g. food insecurity, HIV-related stigma and HIV disclosure). This requires providers with strong interpersonal skills and the competences to provide patient-centred care in a supportive, non-judgemental clinic environment.

Tailored comprehensive support for caregivers should focus on their perceived benefits, on supporting them with problem-solving skills to establish their own routine, strengthening individual motivation, self-efficacy, and encourage access to social support.

## Conclusions

This qualitative study using a realist evaluation approach showed that pellets may mark a major step towards better treatment for HIV positive children. Ease of administration and practical advantages are an added value during the initiation phase of treatment. Further research in non-trial settings may shed light on factors related to providers, services and the health system that contribute to better adherence of such formulations.

## Supporting information

S1 Checklist COREQ checklist. (PDF)
